# Polydopamine surface-modified hyperbranched polymeric nanoparticles for synergistic chemo/photothermal therapy of oral cancer

**DOI:** 10.3389/fbioe.2023.1174014

**Published:** 2023-05-05

**Authors:** Xingyong Yin, Zimu Li, Yi Zhang, Xiaowei Zeng, Qiuxu Wang, Zhigang Liang

**Affiliations:** ^1^ Department of Stomatology, Shenzhen Second People’s Hospital, Shenzhen, China; ^2^ Guangzhou Medical University, Guangzhou, China; ^3^ School of Pharmaceutical Sciences (Shenzhen), Sun Yat-sen University, Shenzhen, China

**Keywords:** cancer nanotechnology, polydopamine, surface modification, oral cancer, synergistic therapy

## Abstract

A novel drug delivery system for the treatment of oral cancer was developed using a facile polydopamine (PDA)-based surface modification and a binding mechanism linking folic acid-targeting ligands. The system was able to achieve the following objectives: loading of chemotherapeutic agents, active targeting, pH responsiveness, and prolonged *in vivo* blood circulation. DOX-loaded polymeric nanoparticles (DOX/H20-PLA@PDA NPs) were functionalized with amino-poly (ethylene glycol)-folic acid (H_2_N-PEG-FA) after coating them with PDA to form the targeting combination, DOX/H20-PLA@PDA-PEG-FA NPs. The novel NPs exhibited drug delivery characteristics similar to DOX/H20-PLA@ PDA NPs. Meanwhile, the incorporated H_2_N-PEG-FA contributed to active targeting, as illustrated in cellular uptake assays and animal studies. *In vitro* cytotoxicity and *in vivo* anti-tumor studies have shown that the novel nanoplatforms exhibit extremely effective therapeutic effects. In conclusion, the multifunctional PDA-modified H20-PLA@PDA-PEG-FA NPs offer a promising chemotherapeutic strategy to improve the treatment of oral cancer.

## Introduction

Approximately 4% of cancers occur in the oral cavity or oropharynx (Nandini et al., 2020). According to statistics, there were about 355,000 cases of oral cancer worldwide in 2018. The number of oral cancer patients in 2020 increased by about 53,260 new cases worldwide from the previous year, and as of the same year, the number of deaths was about 10,750 more than in previous years (Mosaddad et al., 2021). Among oral malignancies, squamous cell carcinoma (SCC) is the most common pathologic classification in the clinical field, and the number of cases of tongue squamous cell carcinoma (TSCC) has been increasing year by year in recent decades (da Silva Souto et al., 2021). At present, the main treatment method for oral cancer is still radical surgery (Marcazzan et al., 2018). Some postoperative patients have to accept postoperative radiotherapy and chemotherapy due to an insufficient pathological evaluation margin and a highly malignant pathological classification (Huang and O'Sullivan, 2013). To remove cancer cells as thoroughly as possible, radical surgery requires a uniform expansion of the resection of the surrounding normal tissue. Therefore, a large number of patients have postoperative maxillofacial deformities, which may lead to the loss of speech, chewing, taste, or other basic functions (Sun et al., 2020). Despite the development of modern medicine, we can repair excised soft tissues with vascularly anastomosed musculocutaneous flap grafts (Girhe et al., 2021), but the appearance and function after the repair are still unsatisfactory. The failure of radiotherapy to target cancer cells leads to non-specific cell death, and many patients experience complications such as mucositis, osteomyelitis, mouth ulcers, and rampant dental caries (Minhas et al., 2017). Chemotherapeutic drugs also have toxic effects on normal cells, and their mode of drug delivery makes them have non-specific tissue distribution in the body, which is easy to cause greater damage to the healthy tissues of the body and produces serious adverse reactions (Zhang et al., 2020).

Breast cancer, lung cancer, cervical cancer, ovarian cancer, prostate cancer, and pancreatic cancer have all been successfully treated in nanomedicine research, and several nanoplatforms have even received clinical approval (Shi et al., 2017; Duo et al., 2018; Zeng et al., 2018; Banstola et al., 2019; Zeng et al., 2019; Yang et al., 2022). Due to the high incidence and poor prognosis of oral squamous cell carcinoma (Li et al., 2020), at present, a variety of effective carrier systems based on nanotechnology have been widely studied as a treatment for oral squamous cell cancer (Gharat et al., 2016). However, there are still no approved nanoplatforms for the clinical treatment of oral cancer. Nanomedicine platforms can also achieve synergistic anticancer therapy, such as chemotherapy combined with photothermal therapy (Liu et al., 2018; Peng et al., 2018; Li et al., 2021; Li et al., 2022), photodynamic therapy (Tao et al., 2018; Mo et al., 2022), immunotherapy (Liang et al., 2020; Jia et al., 2022), photothermal therapy combined with immunotherapy (Zeng et al., 2022), and more. These are conducive to improving the cure rate of malignant tumors. Phototherapies, including photodynamic therapy (PDT) and photothermal therapy (PTT), are non-invasive techniques for cancer treatment. Broadly speaking, phototherapies involve two major steps: first, the delivery of a phototherapeutic agent to tumors, and second, the irradiation of the tumor sites with specific light to activate the phototherapeutic agent. However, most of the reported photosensitizers are highly hydrophobic and cannot be directly applied for treatment purposes (Abbas et al., 2017). Nanomedicine delivery vehicles are promising for loading the photosensitizers into nanoparticles, ensuring that the photosensitizers are stable in an aqueous solution, and also providing better accumulation in tumor tissues through the enhanced permeability and retention (EPR) effect (Zhou et al., 2015; Abbas et al., 2017; Abbas et al., 2018). The vast majority of oral cancers arise from mutations in the oral mucosa or epithelium (Scully and Bagan, 2009), and the lesion is located at the exposed oral site. In superficial local solid tumors, photothermal therapy has been shown to have strong killing effects (Zhang et al., 2019). Therefore, the use of photothermal therapy in the treatment of tumors at exposed oral sites is of great significance for the research. Early stages of oral cancer usually present with persistent oral ulcers, oral masses, or other obvious premalignant lesions (Brandizzi et al., 2008), which contributes to the early detection of lesions. Therefore, the strong killing of local tumor cells by photothermal therapy and targeted chemotherapy can achieve a radical cure for oral cancer in the early stage of the disease and reduce adverse reactions to the body.

The following benefits of polydopamine surface modification make it suitable for different nanoparticle drug carriers: to begin with, PDA membranes contain a dense, cross-linked fabric that increases the stability of nanoparticles (NPs) *in vivo* and prevents early drug release (Park et al., 2014; Zeng et al., 2018; Ci et al., 2019). Furthermore, the abundant quinone groups on the surface of PDA readily react with amino-containing and thiol-containing substances, enabling surface modifications such as binding to PEG and tumor-targeting ligands (Park et al., 2014; Tao et al., 2016; Zhu et al., 2016; Cheng et al., 2017a). Additionally, the PDA surface effectively adsorbs drugs, especially doxorubicin (DOX) (Chang et al., 2016; Cheng et al., 2017b; Liu et al., 2019). Finally, PDA has a high near-infrared photothermal energy conversion efficiency, which suggests that it is a promising and effective phototherapeutic agent (Cheng et al., 2017c; Peng et al., 2018; Zeng et al., 2018).

In addition to passive targeting through EPR effects or active targeting, NPs can increase drug efficacy by improving drug encapsulation and delivery, prolonging cycle half-life, and constantly targeting drug release (Luk and Zhang, 2014; Wei et al., 2016). NPs serve as customizable targeted drug delivery carriers to deliver chemotherapeutic drugs or therapeutic genes to tumor cells. Lower doses of toxic substances can be used because the drug is delivered directly to the target tissue (Poonia et al., 2017). NPs can improve drug stability and control their targeted delivery, thus maintaining constant and uniform concentrations at the lesion site and promoting drug extravasation into the tumor system, thereby reducing side effects. Nanoparticles loaded with photosensitizers can reach the most sensitive subcellular sites, with the ability to treat superficial oral cancers or premalignant lesions (Calixto et al., 2014).

This paper aims to investigate the use of DOX/H20-PLA@PDA-PEG-FA for synergistic chemotherapy and photothermal therapy of oral cancer, which may lead to the development of new oral cancer therapeutic approaches with relatively low side effects.

## Methods

### Characterization of NPs

#### Transmission electron microscopy image

The prepared NPs were resuspended in ethanol, treated with ultrasound to spread them uniformly, and added dropwise to the copper mesh coated with carbon film. After the samples were dried, the surface morphology of the NPs (DOX/H20-PLA NPs, DOX/H20-PLA@PDA-PEG-FA) was observed by transmission electron microscopy.

#### Fourier transform infrared spectroscopy (FT-IR) analysis

The Fourier transform infrared spectra of NPs (DOX/H20-PLA, DOX/H20-PLA@PDA, DOX/H20-PLA@PDA-PEG, DOX/H20-PLA@PDA-PEG-FA) were recorded to analyze the elemental composition of the nanoparticle surface and the chemical modification of the surface.

#### Size distribution and zeta potential (size and zeta potential)

The prepared NPs were resuspended in DI water and treated with ultrasound to spread them uniformly. Dynamic light scattering (DLS) was performed to evaluate the NPs (DOX/H20-PLA, DOX/H20-PLA@PDA, DOX/H20-PLA@PDA-PEG, DOX/H20-PLA@PDA-PEG-FA) in terms of particle size distribution and zeta potential. All experiments were repeated three times independently, and the means were taken.

#### Drug loading content

The supernatant collected from each of the above steps was used to establish the drug loading of the NPs. The drug concentration was calculated by high-performance liquid chromatography (HPLC). For DOX, the mobile phases were phosphate buffer, methanol, and acetonitrile (30:20:50, v/v) at a flow rate of 1 mL/min with 20 μL per injection, and DOX was detected at 233 nm using an ultraviolet-visible (UV-Vis) detector. The *LC* (%) was calculated from the drug standard curve using the following equation.

$$LC\left(\%\right)=\frac{Weight\;of\;DOX\;in\;NPs}{Weight\;of\;NPs}\times 100\%$$ (1)

### Evaluation of the photothermal effect

To evaluate the photothermal properties of the modified nano-drug delivery systems, various NPs (200 μg/mL) and PBS, which were the blank controls, were irradiated under the 808 nm laser with a laser intensity of 1.0 W/cm^2^ for 10 min. Then, the DOX/H20-PLA@PDA-PEG-FA NPs at concentrations of 50, 100, 200, and 500 μg/mL were irradiated under the 808 nm laser with a laser intensity of 1.0 W/cm^2^ for 10 min. To determine the influence of different laser power densities on the photothermal effect, the DOX/H20-PLA@PDA-PEG-FA NPs (200 μg/mL) were irradiated for 10 min under a laser intensity of 0.5, 1.0, 1.5, and 2.0 W/cm^2^, respectively. Finally, to investigate the stability of DOX/H20-PLA@PDA-PEG-FA NPs, they were irradiated for five cycles under a laser intensity of 1.0 W/cm^2^, and each cycle was irradiated for 10 min and then cooled for 10 min. In all experiments, the temperature changes were recorded by a near-infrared imaging camera (Ti 450, Fluke, US), and the temperature curves were plotted.

### Cell viability study

An MTT assay was used to ascertain the cytotoxicity of NPs on SCC-9 cells and TCA-8113 cells. After the SCC-9 cells and the TCA-8113 cells were seeded into a 96-well plate at a concentration of 1 × 10^6^ cells/wells, respectively, they were cultured overnight. The drug-loaded NPs (DOX/H20-PLA@PDA-PEG and DOX/H20-PLA@PDA-PEG-FA) at DOX concentrations of 0, 0.1, 1, 5, and 10 μg/mL were added and cultured for another 24 h and 48 h. Then MTT solution (20 μL, 5 mg/mL) was added to each well, and the cells were cultured for an additional 4 h. The media containing MTT was aspired after 4 h. Then the DMSO was dropped into each well and the crystals were allowed to dissolve for 2 h in the dark at 37°C. The optical density value of each well was detected using a microplate reader at a wavelength of 490 nm. The control group (drug-free H20-PLA@PDA-PEG-FA NPs) represented zero absorbance. Cell viability was evaluated by MTT at each time point. In addition, the toxicity of the above-mentioned drug-loaded NPs to the target cells after laser irradiation was compared. Cell viability data compared to control subjects were examined by curve fitting.

### Animals and tumor model

Female nude mice (BALB/c-nude, 4–5 weeks old) were purchased from the Guangdong Medical Laboratory Animal Center (China), and all *in vivo* experimental protocols were approved by the Institutional Animal Care and Use Committee of Sun Yat-sen University (Approval No. SYSU-IACUC-2022-000836). After 1–2 weeks of culture in a specific pathogen-free (SPF) grade laboratory chamber, each mouse (18–20 g) was injected with 100 μL suspended SCC-9 cells (2 × 10^6^ cells) in PBS to establish the cell xenograft model. The tumor volume (V) was measured with a vernier caliper and then calculated by aliquot: V = A×B^2^/2, where A and B refer to the length and width of the tumor, respectively.

### *In Vivo* anti-tumor efficacy

After the tumor volume was increased to 200 mm^3^, the mice were randomly divided into 6 groups (n = 5). Intravenous saline was administered as a control. Every 4 days (0, 4, 8, 12, 16), each group of nude mice was injected separately with 100 μL saline, drug-free H20-PLA@PDA-PEG-FA, DOX, and drug-loaded NPs (DOX/H20-PLA@PDA-PEG, DOX/H20-PLA@PDA-PEG-FA) via the tail vein at a DOX density of 10 mg/kg. In addition, the photothermal group was irradiated after each injection of DOX/H20-PLA@PDA-PEG-FA NPs. The tumor volume was recorded every 1 day with a caliper, and its weight was measured once. Mice were sacrificed after 20 days of treatment, and their tumor tissues were isolated and weighed. Major organs (heart, lung, liver, spleen, and kidney) and tumors were then collected and fixed in 10% neutral formalin for histological analysis. After paraffin embedding, tissues, and samples were cut into approximately 4 μm sections and analyzed by light microscopy after staining with hematoxylin and eosin (H&E).

## Results and discussion

### Synthesis of polymeric NPs

**FIGURE 1:**
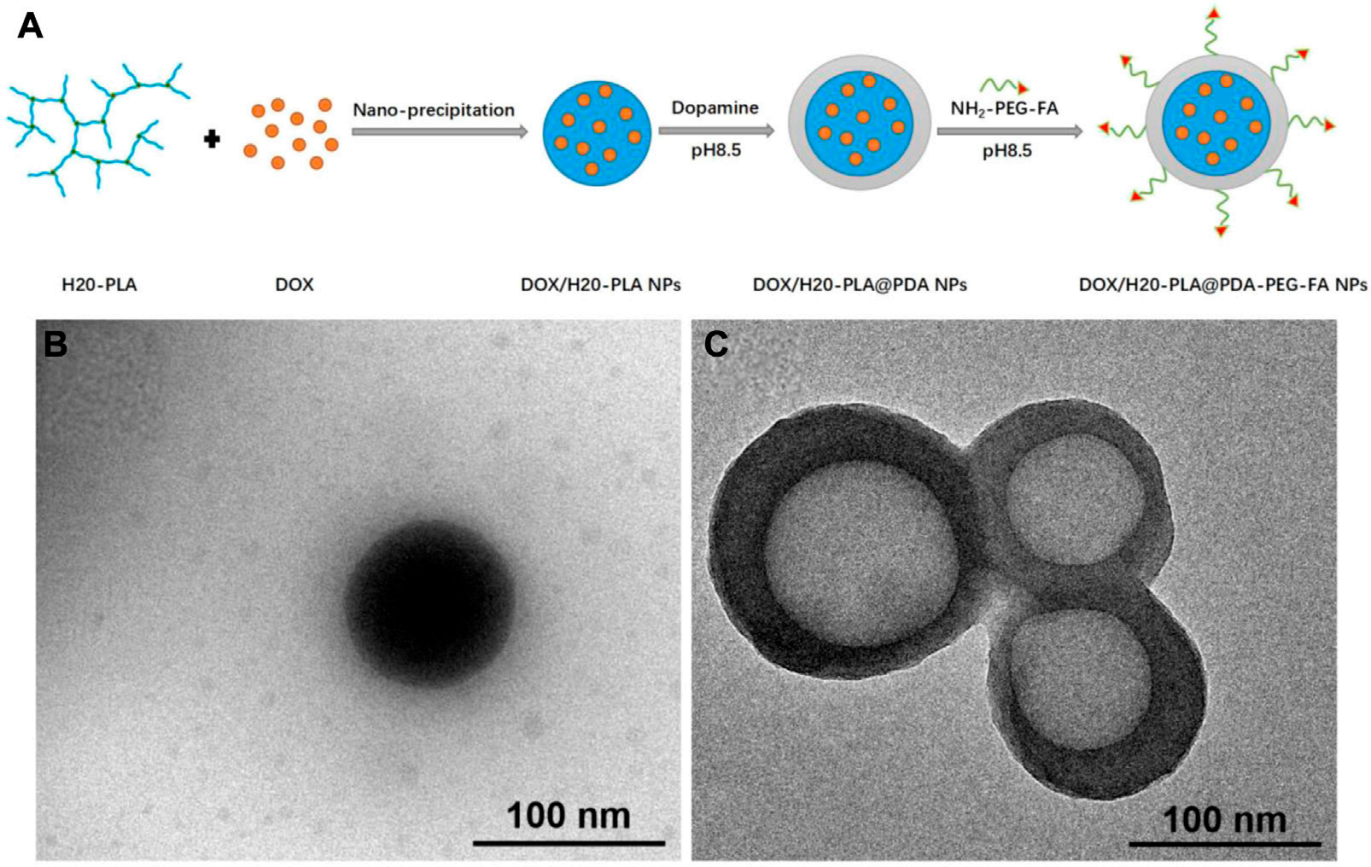
**(A)** Schematic illustration of the preparation procedure of the targeted DOX/H20-PLA@PDA-PEG-FA NPs, **(B)** TEM images of DOX/H20-PLA NPs, **(C)**DOX/H20-PLA@PDA-PEG-FA NPs.

The preparation of the dendritic copolymer H20-PLA by ring-opening polymerization is shown in Supplementary Figure S1. As shown in Figure 1A, the preparation process of the target NPs mainly included the loading with the broad-spectrum anticancer drug doxorubicin (DOX), the surface modification of polydopamine, and the attachment of the targeting ligands. Under weakly alkaline conditions, dopamine monomers were oxidized to quinones and polymerized to form polydopamine to adhere to the surface of the nanoparticles, which was useful to achieve both targeting ligand attachment and anticancer drug adsorption with good photothermal efficiency (Scully and Bagan, 2009). H_2_N-PEG-FA was used as the targeting ligand and attached to the polydopamine on the nanoparticle surface via the Michael addition reaction under alkaline conditions. The DOX/H20-PLA@PDA-PEG-FA NPs were obtained as a targeted nano-delivery system for combined chemotherapy and photothermal therapy.

### Characterization of polymeric NPs

According to Figures 1B,C and Supplementary Figure S2, both DOX/H20-PLA NPs and DOX/H20-PLA@PDA-PEG-FA NPs were observed as smooth nanospheres under transmission electron microscopy. The DOX/H20-PLA@PDA-PEG-FA NPs had an apparent core-shell structure, demonstrating that the PDA layer was deposited on the surface of the NPs (Liu et al., 2019). As evidence, Supplementary Figure S3 showed that: 1) the FT-IR spectra of all NPs can be found with absorption peaks at 1760 cm^−1^, representing the carbonyl group in H20-PLA; 2) the absorption peak at 1,510 cm^−1^ is attributed to the bending vibration of N-H; and 3) the broad absorption between 3,600 cm^−1^ and 3,300 cm^−1^ is due to the stretching vibration of the N-H/O-H group. The above results suggest that the surface of the NPs is modified with PDA in addition to PEG or PEG-FA.

Both the size and zeta potential of NPs are essential for their stabilization and EPR effects (Bertrand et al., 2014; Sykes et al., 2014). NPs in the 10–200 nm diameter range are most likely to be taken up by tumor tissue through the EPR effects (Sykes et al., 2014; Liu et al., 2019). Dynamic light scattering (DLS) was used to determine the size and size distribution of the nanoparticles. As illustrated in Supplementary Table S1 and Supplementary Figure S4, the average diameters of the drug-loaded NPs (DOX/H20-PLA NPs, DOX/H20-PLA@PDA NPs, DOX/H20-PLA@PDA-PEG NPs and DOX/H20-PLA@PDA-PEG-FA NPs) were approximately in the range of 100–160 nm, respectively. The low polydispersity index (PDI <0.2) indicates that they have a relatively uniform size distribution, which facilitates drug delivery *in vivo*. The zeta potentials of NPs were negative, which facilitated prolonged cycling before tumor tissue enrichment (Zhu et al., 2015; Linlin et al., 2016). After surface modification of DOX/H20-PLA NPs with polydopamine, the zeta potential was still negative. This is probably explained by the deprotonation of the phenolic hydroxyl group of polydopamine at neutral pH (Yu et al., 2010; Kim et al., 2014). The modified hydrophilic PEG segment reduced the absolute value of the zeta potential due to the surface charge shielding effect (Chen et al., 2017). According to the test results, the drug loading of the NPs was greater than 8.4%, indicating that the nanoparticles’ surface modification did not significantly reduce the drug loading and the drug-loaded NPs had a good level of drug loading.

When NPs are temporarily stored after preparation, they appear to aggregate depending on the decrease in the absolute value of the zeta potential. Maintaining the stability of NPs is essential for therapeutic efficacy. Their average size and zeta potential were examined every 10 days after preparation to observe the stability of the drug-loaded NPs under storage conditions. As shown in Supplementary Figure S5, the size and zeta potential of the NPs did not change significantly during storage (90 days), and the data suggest that the NPs are quite stable.

#### Photothermal effect and drug release profiles of NPs

**FIGURE 2:**
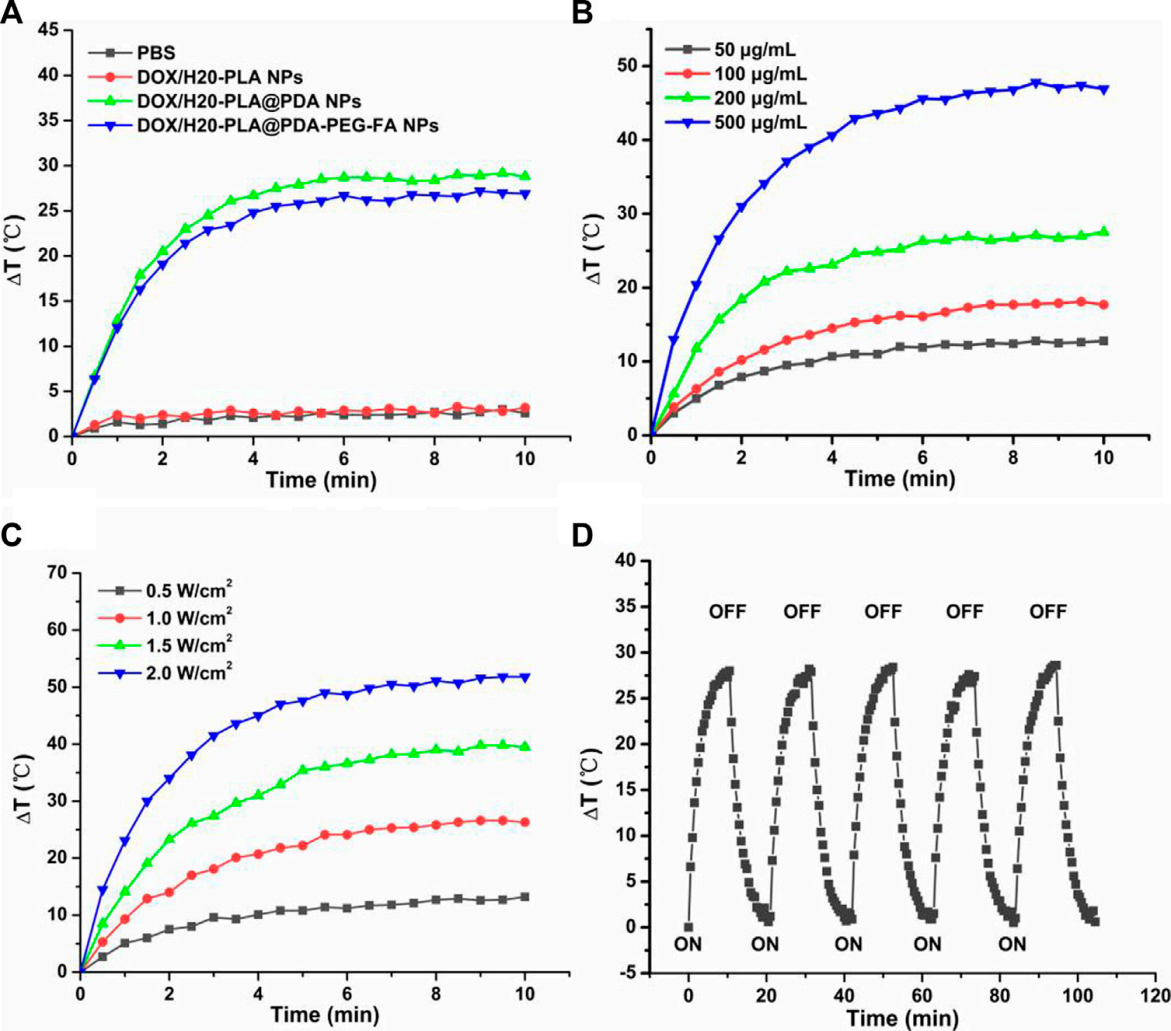
**(A)** Temperature variation curves of the aqueous dispersions of PBS, DOX/H20-PLA NPs, DOX/H20-PLA@PDA NPs, and DOX/H20-PLA@PDA-PEG-FA NPs (200 μg/mL) exposed to an 808 nm laser at a power density of 1.0 W/cm^2^ for 10 min. **(B)** Temperature variation curves of DOX/H20-PLA@PDA-PEG-FA NPs with different concentrations. **(C)** Temperature variation curves of DOX/H20-PLA@PDA-PEG-FA NPs (200 μg/mL) under different power intensities. **(D)** Temperature variation curves of DOX/H20-PLA@PDA-PEG-FA NPs (200 μg/mL) with five cycles of consecutive laser irradiation.

As shown in Figure 2A, the temperature of DOX/H20-PLA@PDA NPs and DOX/H20-PLA@PDA-PEG-FA NPs rapidly increased by more than 25°C within 10 min under irradiation at an intensity of 1.0 W/cm^2^. In contrast, the temperature of PBS and DOX/H20-PLA NPs under the same conditions showed no significant change. The results indicate that the drug-loaded polymer nanoparticles (DOX/H20-PLA NPs) do not have photothermal properties by themselves but have photothermal conversion properties after their PDA surface modification. This is also consistent with many previous studies showing that PDA has obvious NIR absorption and better photothermal conversion efficiency. In addition, as shown in Figures 2B,C, both power intensity and nanoparticle concentration also affected the photothermal efficiency of NPs. For example, as the power intensity of the layer increased from 0.5 to 2.0 W/cm^2^, the temperature of 200 μg/mL DOX/H2O-PLA@PDA-PEG-FA NPs increased rapidly, or as the nanoparticle concentration increased from 50 to 500 μg/mL, the temperature of DOX/H20-PLA@PDA-PEG-FA NPs at the laser intensity of 1.0 W/cm^2^ also increased rapidly. Therefore, DOX/H20-PLA@PDA-PEG-FA NPs exhibited photothermal efficiency depending on laser power intensity and concentration. 200 μg/mL DOX/H20-PLA@PDA-PEG-FA NPs were irradiated for 5 on/off cycles at a laser intensity of 1.0 W/cm^2^. In each cycle, irradiation was performed for 10 min followed by cooling for 10 min. Through cycling experiments (Figure 2D), we found that the temperature change of DOX/H20-PLA@PDA-PEG-FA NPs is not significant, which indicates that DOX/H20-PLA@PDA-PEG-FA NPs have good photothermal stability. In conclusion, DOX/H20-PLA@PDA-PEG-FA NPs may become a promising potential photothermal cancer therapy.

**FIGURE 3:**
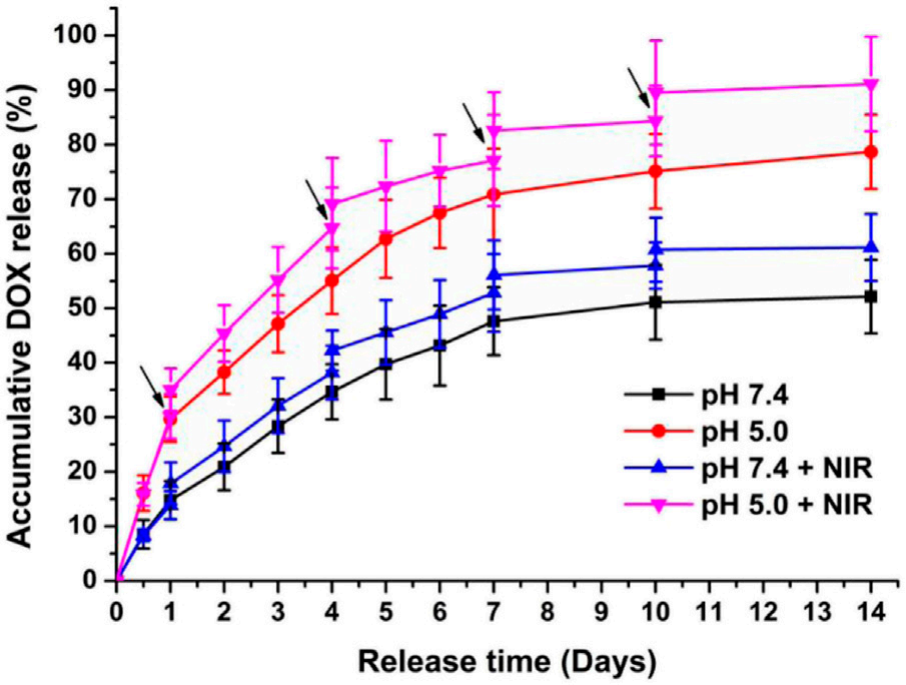
*In vitro* drug release profiles of DOX/H20-PLA@PDA-PEG-FA NPs at different pH with or without NIR laser irradiation (808 nm, 1.0W/cm^2^). ↓: NIR irradiation for 10 min.

The NPs will be exposed to different microenvironments whose pH values are quite different during *in vivo* delivery, such as pH∼7.4 in the bloodstream and pH∼5.0 in the lysosome. The drug release curves of DOX/H20-PLA@PDA-PEG-FA NPs in the medium of pH = 7.4/5.0 were shown in Figure 3. The results indicated that the DOX release curves of DOX/H20-PLA@PDA-PEG-FA NPs showed obvious pH responsiveness and the laser irradiation dependence. All of the above drug release curves displayed an explosive release of DOX in the primary stage and then entered a slow-release stage. After 14 days, the final drug release levels were approximately 75% and 50% at pH 5.0 and pH 7.4, respectively, which was probably attributable to the shedding of the PDA film from the acidic environment. This facilitated the release of DOX. Furthermore, the DOX/H20-PLA@PDA-PEG-FA NPs enhanced the drug release by about 10%–15% each time under laser irradiation, which may be attributed to the photothermal effect of PDA. It may reduce premature DOX release during cycling and increase specific release in the acidic tumor microenvironment due to this pH responsiveness and the laser radiation dependence of NPs. This also reduces the side effects of the drug as well as modulates the volume of drug release in the cells. Therefore, this drug delivery system may be desirable and promising.

#### Cellular uptake of fluorescent NPs

**FIGURE 4:**
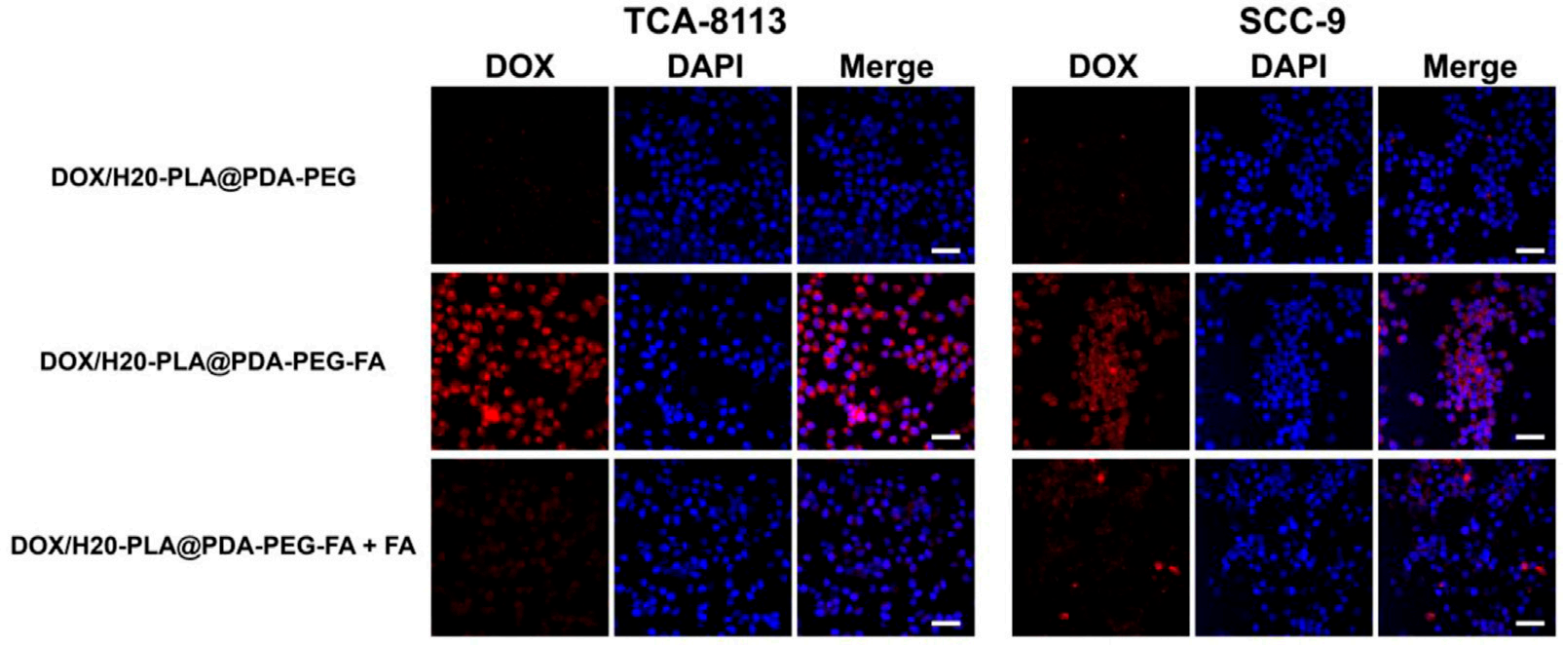
Confocal laser scanning microscopy (CLSM) images of TCA-8113/SCC-9 cells after incubation with NPs for 2 h. Blue: DAPI-stained nucleus. Scale bar = 20 μm.

To investigate the uptake of NPs by tumor cells, these (TCA-8113 cells/SCC-9 cells) were labeled with DAPI and analyzed by confocal laser scanning microscopy (CLSM). Figure 4 shows that DOX/H20-PLA@PDA-PEG NPs were not well taken up by the two aforementioned tongue squamous carcinoma cells, as no red fluorescence was observed in the non-aptamer group. On the contrary, the red fluorescence around the blue fluorescence of the nucleus could be seen in the aptamer group, indicating that a large amount of DOX/H20-PLA@PDA-PEG-FA NPs entered the tumor cytoplasm, and no significant difference was observed in the two types of tongue squamous carcinoma cells. To verify whether the cellular uptake mechanism in the active targeting group was mediated by aptamers or not, both active targeting NPs and free aptamers were added to the cell cultures in the control group. According to Figure 4, the addition of aptamers significantly reduced the red fluorescence, suggesting that the uptake of active targeting NPs was associated with aptamer-mediated endocytosis.

### Effect of NPs on cell viability

The cytotoxicity of the nanoparticles to SCC-9 cells and TCA-8113 cells *in vitro* was assessed using MTT assays and compared with the toxicity of the same nanoparticles to the target cells after laser irradiation. Drug-free H20-PLA@PDA-PEG-FA NPs were used to evaluate the toxicity of the drug vehicle to cells.

**FIGURE 5:**
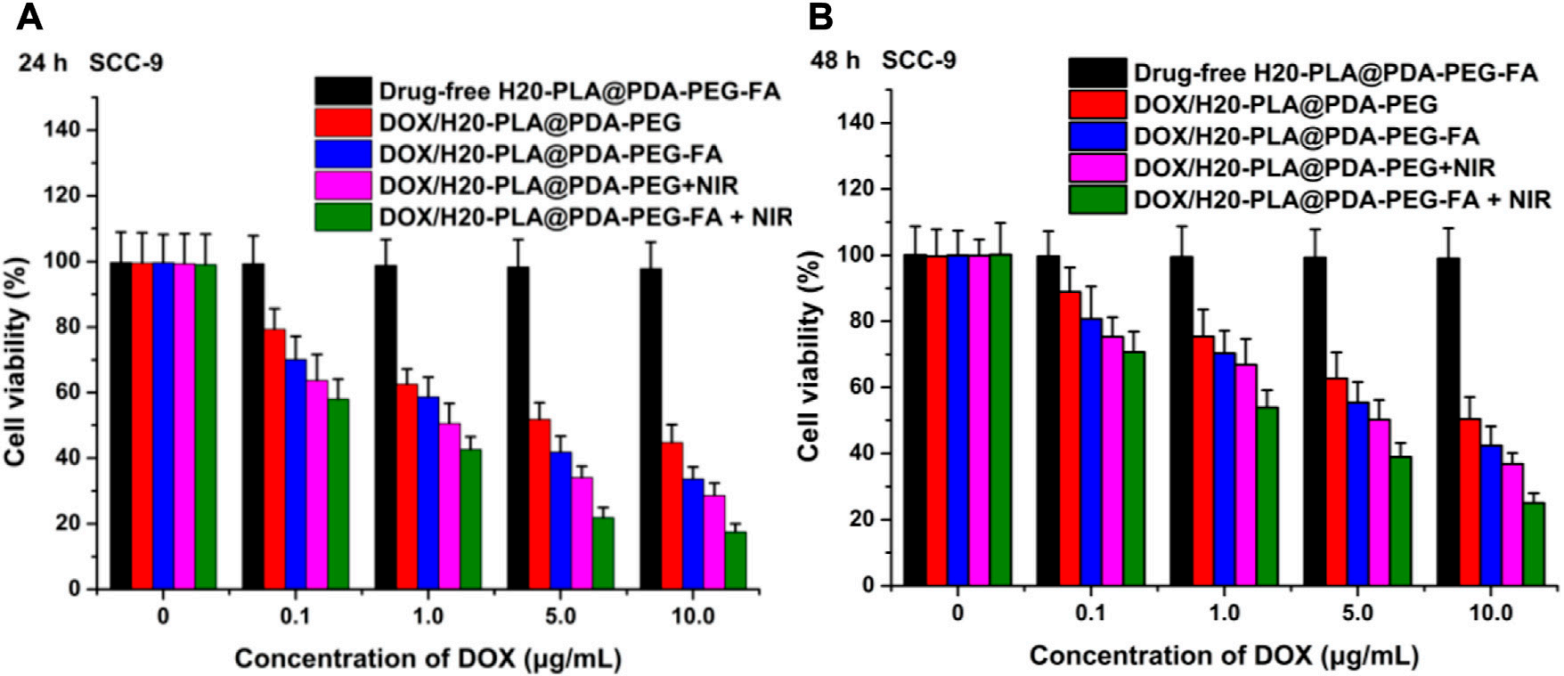
Viability of SCC-9 cells cultured with drug-loaded NPs (DOX/H20-PLA@PDA-PEG NPs, DOX/H20-PLA@PDA-PEG-FA NPs) with or without NIR laser irradiation (808 nm, 1.0W/cm^2^) compared with drug-free NPs at the same dose for **(A)** 24 h and **(B)** 48 h (t-test, *p < .05, **p < .01, ***p < .001).

**FIGURE 6:**
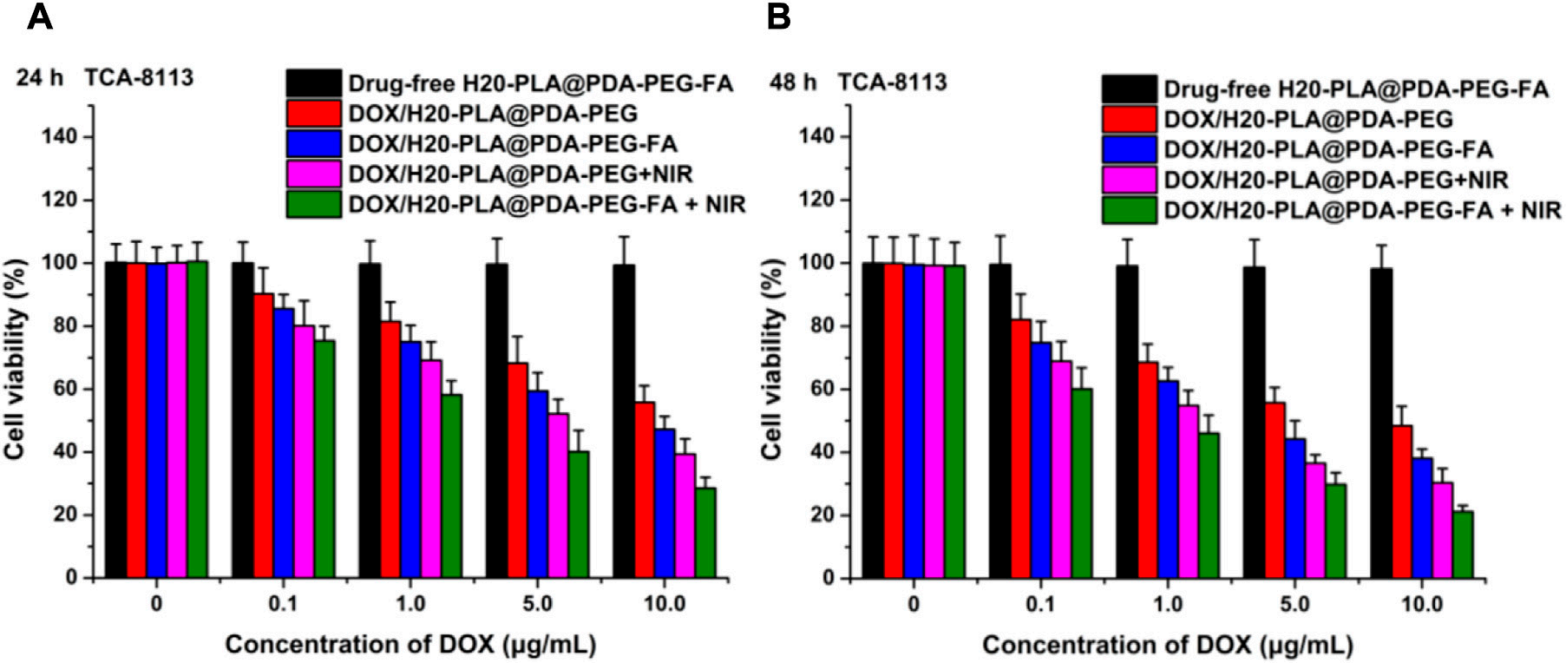
Viability of TCA-8113 cells cultured with drug-loaded NPs (DOX/H20-PLA@PDA-PEG NPs, DOX/H20-PLA@PDA-PEG-FA NPs) with or without NIR laser irradiation (808 nm, 1.0W/cm^2^) compared with drug-free NPs at the same dose for **(A)** 24 h and **(B)** 48 h (t-test, *p < .05, **p < .01, ***p < .001).

The cytotoxicity of the nano-drug carriers used in this experiment was evaluated by measuring the cell viability of SCC-9 and TCA-8113 cells after 24 h (Figure 5A; Figure 6A) and 48 h (Figure 5B; Figure 6B) of therapy with drug-free H20-PLA@PDA-PEG-FA NPs. According to the results, the cells treated with drug-free H20-PLA@PDA-PEG-FA NPs exhibited a survival rate close to 100% under all conditions, indicating that the vector material was essentially non-toxic to both cells.

The following results were obtained: 1) the toxicity of all drug-loaded NPs to TCA-8113 cells intensified with increasing drug concentration and also with increasing duration of action (Figure 6); 2) the toxicity of all drug-loaded NPs to SCC-9 cells intensified with increasing drug concentration, but the cytotoxicity decreased after the duration of action beyond 24 h (Figure 5); 3) under the same treatment time and the same drug concentration, the cytotoxicity of drug-loaded NPs with targeting ligands was significantly higher than that of NPs without targeting ligands (Figure 5; Figure 6), indicating that the active targeting mechanism mediated by folic acid was more helpful for drug-loaded NPs to kill tumor cells than the passive accumulation mechanism; 4) under the same treatment time and the same drug concentration, the cytotoxicity of different drug-loaded NPs significantly increased under NIR laser irradiation (Figure 5; Figure 6), which was consistent with the laser irradiation-dependent toxicity of the drug-loaded nanoparticles in the *in vitro* drug release experiment; 5) for TCA-8113 cells, the experimental group treated with DOX/H20-PLA@PDA-PEG-FA NPs at a DOX concentration of 10.0 μg/mL for 48 h and simultaneously treated with laser irradiation exhibited the lowest cell viability, i.e., the highest cytotoxicity, among all the experimental groups; 6) for SCC-9 cells, the group treated with DOX/H20-PLA@PDA-PEG-FA NPs at a concentration of 10.0 μg/mL for 24 h and simultaneously treated with laser irradiation exhibited the lowest cell survival rate, i.e., the highest cytotoxicity, among all the experimental groups. In conclusion, the active targeting mechanism combined with synergistic photothermal treatment improved the cytotoxicity of the tumor cells.

### *In Vivo* anti-tumor efficacy

Based on the cytotoxicity assay, the *in vivo* anti-tumor effect of drug-loaded nanoparticles was further investigated to verify the *in vivo* tumor suppressive effect. We used injected saline as a blank control and injected drug-free H20-PLA@PDA-PEG-FA NPs, DOX, drug-loaded NPs (DOX/H20-PLA@PDA-PEG NPs, DOX/H20-PLA@PDA-PEG-FA NPs), and DOX/H20-PLA@PDA-PEG-FA NPs + NIR (as photothermal treatment). Injections were given every 4 days during the 20-day treatment cycle, and the photothermal team was treated with laser irradiation after 24 h. Tumor volume measurements using vernier calipers and weighing of nude mice were performed every 1 day. The nude mice were sacrificed 20 days later, and the tumor tissue was isolated.

**FIGURE 7:**
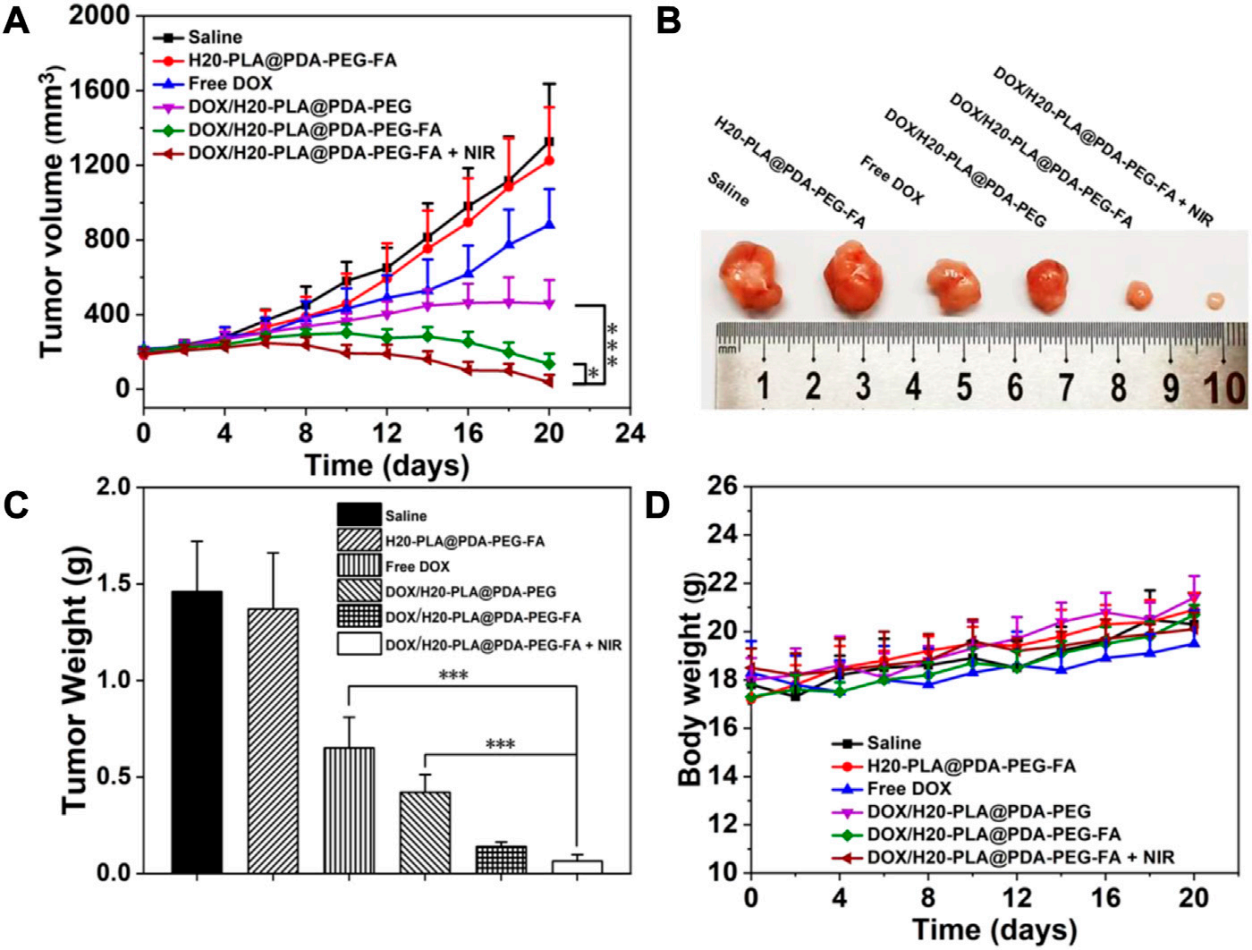
**(A)** Changes in tumor volumes treated with different NPs. **(B)** Morphology of representative tumors removed from the sacrificed mice treated with different NPs. **(C)** Tumor weights of each group treated with different NPs. **(D)** Changes in body weight of mice treated with different NPs. Data are expressed as mean ± SD (n = 5). ∗*p* < 0.05 and ∗∗∗*p* < 0.001.

The results were as follows: 1) there was no significant difference in tumor volume and weight between the saline and drug-free NPs groups (Figures 7A–C), indicating that drug-free NPs were not lethal to the tumor tissue; 2) the tumor volume and weight in the naked drug group were larger than all experimental groups containing drug-loaded NPs (Figures 7A–C), which may be due to the hydrophilic PEG modification on the surface of NPs, which helped to reduce the reticuloendothelial tissue clearance, resulting in a greater effective drug accumulation at the tumor site in the drug-loaded NPs group than in the naked drug group; 3) the tumor volume in the DOX/H20-PLA@PDA-PEG-FA NPs group during the experimental cycle was basically smaller than that in the DOX/H20-PLA@PDA-PEG NPs group (Figure 7A), and finally, the DOX/H20-PLA@PDA-PEG -FA NPs experimental group also had a significantly lower tumor tissue weight than the DOX/H20-PLA@PDA-PEG NPs experimental group (Figure 7C), indicating that the targeting ligand folic acid binds to the folic acid receptor overexpressed on the surface of tumor cells and facilitates the uptake of drug-loaded nanoparticles by tumor cells; 4) the volume of tumors in the experimental group treated with DOX/H20-PLA@PDA-PEG-FA NPs + NIR gradually decreased during the treatment cycle (Figure 7A), indicating that the targeted nano-delivery system combined with photothermal treatment could significantly inhibit tumor growth; 5) the tumor volume of the experimental group treated with DOX/H20-PLA@PDA-PEG-FA NPs basically did not increase during the treatment cycle (Figure 7A), indicating its effect in inhibiting tumor growth; 6) in the same time period, the body weight of nude mice in each experimental group was basically not different from that of the saline group (Figure 7D), indicating that the drug-loaded NPs had no obvious toxic side effects on nude mice. In conclusion, the targeted nano-drug delivery system based on polydopamine in combination with photothermal therapy can inhibit tumor growth with essentially no toxic side effects and has good prospects for tumor treatment.

#### Histological analysis

**FIGURE 8:**
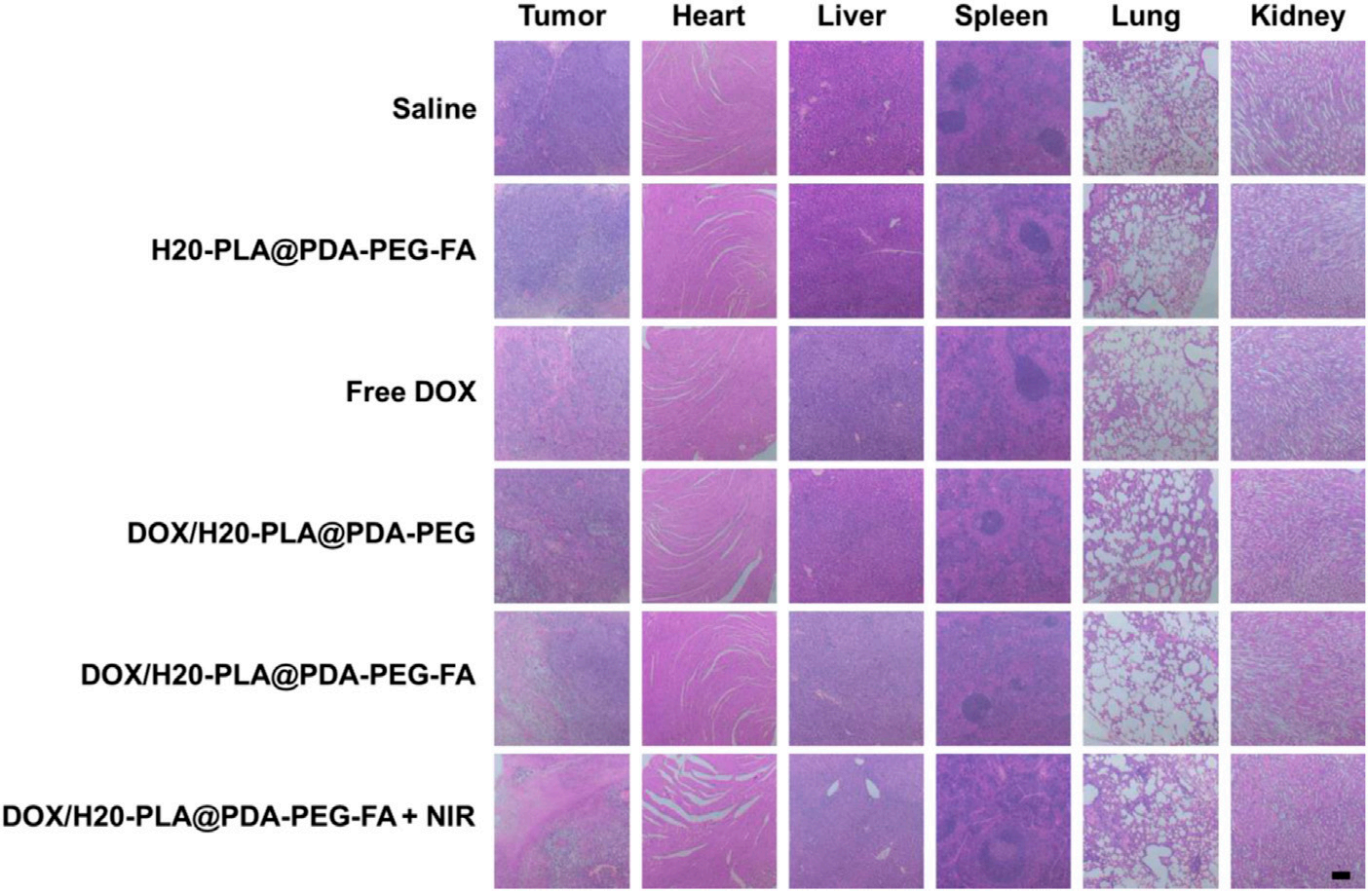
Representative H&E stained images of tumors and major organs (heart, liver, spleen, lung, and kidney) of each group treated with different NPs. Scale bar = 200 μm.

The nude mice were sacrificed and their major organs (heart, lung, liver, spleen, and kidney) collected, and the effects of drug-loaded nanoparticles on the major organs were further investigated using a tissue section analysis assay. As shown in Figure 8, there was no significant damage to the major organs and tumor tissues in both the saline control group and the nude mice with drug-free nanoparticles, which means that the drug-free nanoparticles were not cytotoxic. For the drug-loaded nanoparticle group, there was no significant damage to the major organ sections, but the tumor tissue sections had a wide range of apoptosis and damage: large areas of cell necrosis were observed in the tumor tissue sections treated with DOX/H20-PLA@PDA-PEG-FA + NIR, indicating that it caused more severe damage to the tumor and improved tumor suppression efficacy.

In conclusion, the tumor-inhibiting effect of drug-loaded nanoparticles *in vivo* and the results of cytotoxicity experiments are mutually consistent. The drug-loaded nanoparticles essentially inhibited tumor growth without causing damage to major organs. Therefore, DOX/H20-PLA@PDA-PEG-FA + NIR therapy may be a safe and effective novel tumor treatment modality.

## Conclusion

The DOX/H20-PLA@PDA-PEG-FA NPs used in this experiment were prepared according to the following steps: DOX loading, surface modification with polydopamine, and targeting ligand attachment. The surface modification of the nanoparticles with a polydopamine molecular layer and targeting ligands promoted the passive accumulation and active targeting of the nanoparticles in the tumor tissue, respectively, which further improved the distribution of the nanoparticles *in vivo*. Due to the photothermal effect and the pH sensitivity of the PDA films, drug release was accelerated in the acidic tumor microenvironment under laser irradiation. *In vitro* results show that the NPs display non-cytotoxicity and high biocompatibility. In addition, the NPs showed outstanding chemotherapeutic-photothermal synergy under laser irradiation, probably due to their excellent photothermal conversion properties. In conclusion, the NPs exhibit the following characteristics: long-lasting cycling *in vivo* (PEG), active targeting (FA), pH responsiveness (PDA), and chemotherapeutic drug loading and release. This novel polydopamine-surface-modified nanoplatform offers a new direction in oral cancer therapy with significant potential for tumor therapy.

## Data Availability

The original contributions presented in the study are included in the article/Supplementary Material, further inquiries can be directed to the corresponding authors.
