# Supplementary material for: Polydopamine surface-modified hyperbranched polymeric nanoparticles for synergistic chemo/photothermal therapy of oral cancer
Source: Front Bioeng Biotechnol. 2023 May 5;11:1174014. doi: 10.3389/fbioe.2023.1174014 (PMC10197810; doi:10.3389/fbioe.2023.1174014)
Supplement: Supplementary file 1 [file DataSheet1.docx]

***Supporting Information***

**Polydopamine Surface-Modified Hyperbranched Polymeric Nanoparticles for the Chemo/Photothermal Synergistic Therapy of Oral Cancer**

Xingyong Yin^1,2^, Zimu Li^3^, Yi Zhang^3^, Xiaowei Zeng^3*^, Qiuxu Wang^1*^ and Zhigang Liang^1*^

*^1^ Department of Stomatology, Shenzhen Second People's Hospital, Shenzhen, China*

*^2^ Guangzhou Medical University, Guangzhou, China*

*^3^ School of Pharmaceutical Sciences (Shenzhen), Sun Yat-sen University, Shenzhen, China*

^*^ Corresponding authors.

E-mail: [zengxw23@mail.sysu.edu.cn](mailto:zengxw23@mail.sysu.edu.cn) (X. Zeng)

E-mail: [wangqx@sj-hospital.org](mailto:wangqx@sj-hospital.org) (Q. Wang)

E-mail: liangzhigang@yeah.net (Z. Liang)

### 1. Materials

D-α-tocopheryl polyethylene glycol 1000 succinate (TPGS), dopamine hydrochloride, and 3-(4,5-dimethyl-2-thiazolyl)-2,5-diphenyl-2-H-tetrazolium bromide (MTT) were purchased from Sigma-Aldrich (St. Louis, MO, USA). Dendritic copolymer H20-PLA (*M*_w_≈20,000) was provided by the School of Pharmaceutical Sciences (Shenzhen), Sun Yat-sen University, and which synthetic formula is shown in **Figure S1**. Doxorubicin (DOX) hydrochloride was purchased from Dalian Meilun Biology Technology Co., Ltd. (Dalian, China). Amino-terminated PEG-FA (H_2_N-PEG-FA, Mw = 2000) were provided by Shanghai Yare Biotech, Inc. (Shanghai, China). Acetonitrile and dichloromethane were purchased from Sinopharm Chemical Reagent Co., Ltd. (Guangzhou, China). All other chemicals of the highest quality were commercially available and used without further purification. Human oral cancer cell line TCA-8113 cells and SCC-9 cells were purchased from American Type Culture Collection (ATCC, Rockville, MD).

### 2. Preparation of Drug-Loaded NPs

The drug-loaded NPs were prepared by the nanoprecipitation method. 200 mg of H20-PLA and 20 mg of DOX were dissolved in 16 ml of the organic solvent acetone, and then added dropwise to 200 ml of 0.03% TPGS aqueous solution and stirred overnight. Then centrifuged above solution was at 20,000 rpm for 20 min. The supernatant was removed and the precipitate was washed three times with DI water. The obtained DOX/H20-PLA NPs were freeze-dried and used later.

### 3. Modification of nanoparticles

Surface modification of polydopamine: Weigh an appropriate amount of the drug-loaded NPs prepared in 2, and resuspend them in Tris buffer (10 mmol/L, pH=8.5) which is equipped with the mixture at a concentration of 1 mg/mL. And weigh an appropriate amount of dopamine hydrochloride into the above solution to make the final concentration of dopamine 0.5 mg/mL. The above mixture was stirred at room temperature in the dark for 5 min, and centrifuged at 20,000 rpm for 20 min. The supernatant was removed and washed three times with DI water to remove unencapsulated polydopamine. The obtained DOX/H20-PLA@PDA NPs were freeze-dried and prepared for use.

### 4. Ligand Attachment

Weigh an appropriate amount of NPs prepared in 3, and suspend them in Tris buffer (10 mmol/L, pH=8.5) which is equipped with the mixture at a concentration of 1 mg/mL. And add an appropriate amount of targeting ligand was added to the above solution to give a final concentration of 2 mg/mL of targeting ligand. Then stirred at room temperature with protection from light for 5 min, and centrifuged at 20,000 rpm for 20 min. The supernatant was removed, and the precipitate was washed three times with DI water. After freeze-drying, obtain DOX/H20-PLA@PDA-PEG-FA NPs modified with targeting ligand and polydopamine.

### 5. *In Vitro* Drug Release

In vitro drug release from NPs was monitored by dialysis and centrifugation. Generally speaking, 5 mg of drug-loaded NPs (DOX/H20-PLA@PDA-PEG-FA NPs) were dispersed into PBS (1 mL) containing 0.1% Tween-80. The mixture was then poured into a dialysis bag. The dialysis bag was immersed in 15 ml of PBS release medium (pH 7.4 or 5.0) in a 50 mL centrifuge tube. Next, the tube was placed in an orbital water bath and shaken at 37℃. The release medium in the tube was replaced with fresh medium at the designated intervals and the medium containing DOX was filtered for subsequent HPLC analysis. After the removal of the aqueous phase, the organic phase was evaporated by introducing nitrogen. Then 1 mL of mobile phase was added to dissolve the drug and HPLC was performed with a 20 μL solution. Drug concentrations were calculated according to the standard curve and in vitro release curves were plotted. In addition, the 808 nm NIR laser with a power density of 1.0 W/cm^2^ was applied for 10 min to compare the in vitro drug release before and after irradiation.

### 6. Cellular Uptake of Fluorescent NPs

TCA-8113 cells / SCC-9 cells were seeded into six-well plates at a concentration of 1×10^6^ cells/wells respectively and cultured overnight. NPs (200 μg/mL) added to the plates need to co-culture with cells for 2 h. The cells were washed 3 times with PBS and fixed with 4% paraformaldehyde for 15 min. Then they were stained with DAPI for 10 min and washed 3 times with PBS for 5 min each time. Confocal laser scanning microscopy (CLSM) was used to observe the cellular uptake of NPs. The excitation wavelengths for the blue and red channels are respectively 340 and 488 nm.

### 7. Statistical Analysis

SPSS 25.0 software was used for data analysis. All values of the experiments are performed as the mean ± standard deviation (SD). T-tests were used to compare between two groups and P<0.05 was considered statistically significant and the test significance level (α) was 0.05.

**
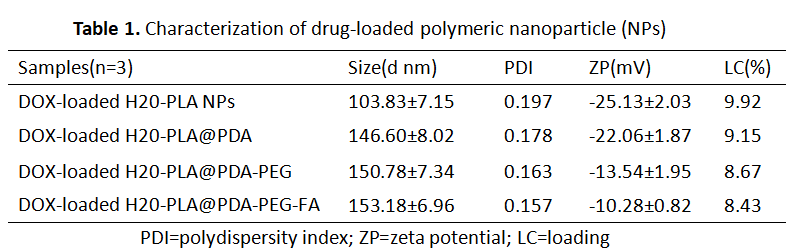
Table S1.** Characterization of drug-loaded polymeric nanoparticles (NPs)





**Figure S1.** The synthetic formula for H20-PLA.


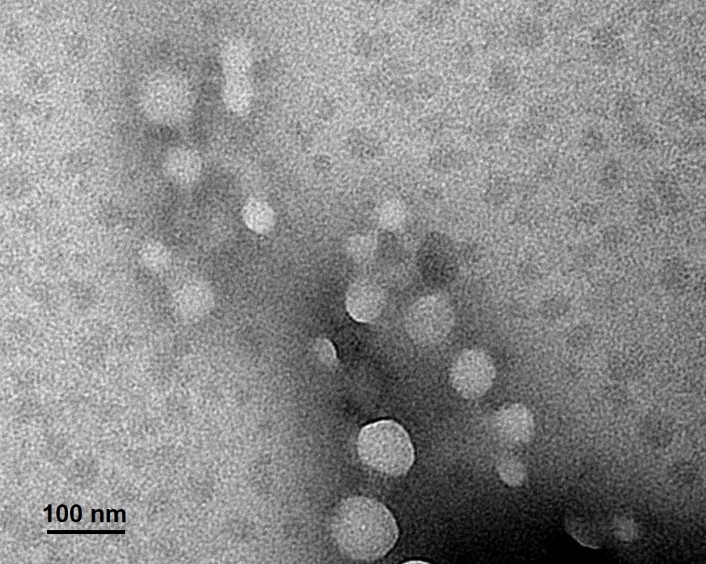


**Figure S2.** TEM images of DOX/H20-PLA NPs.





**Figure S3.** FT-IR spectra of H20-PLA NPs, H20-PLA@PDA NPs, H20-PLA@PDA-PEG NPs and H20-PLA@PDA-PEG-FA NPs.


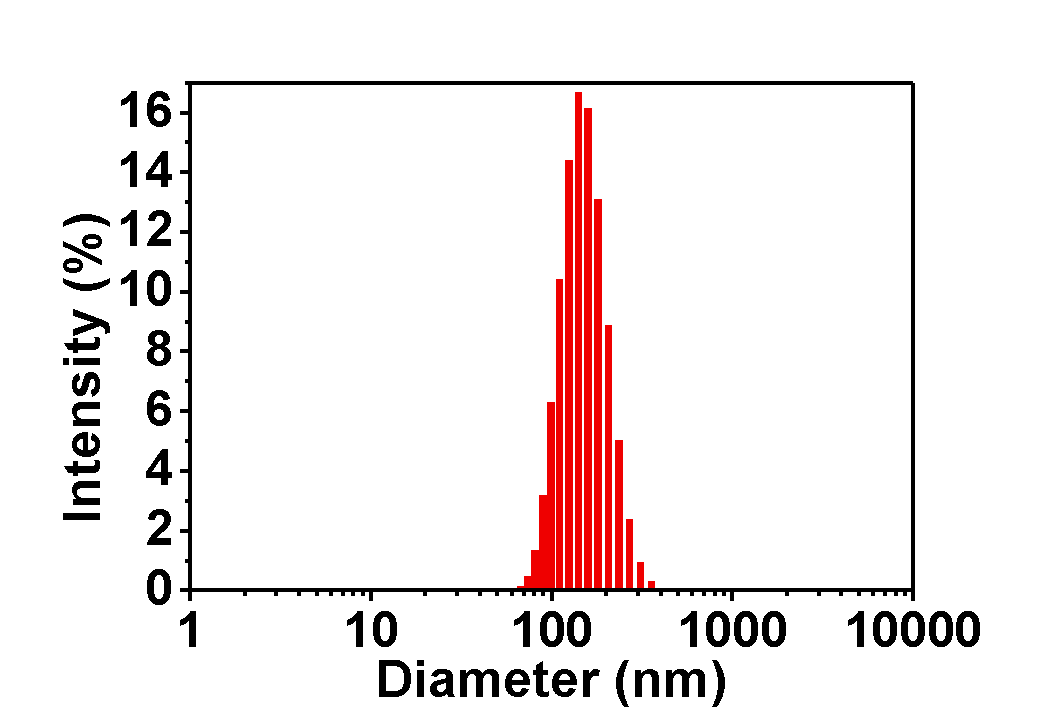


**150.78 nm**

**CC**


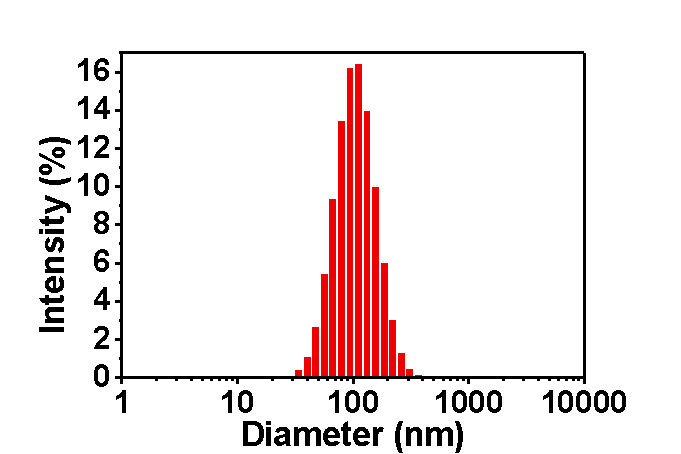


**103.83 nm**

**AC**


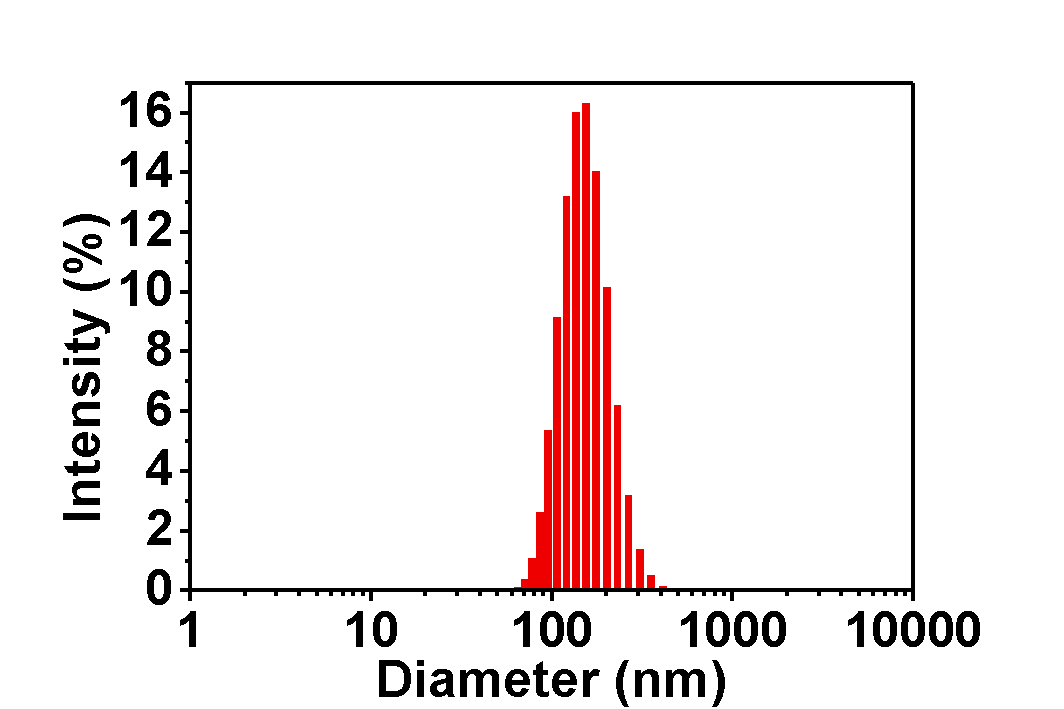


**146.60 nm**

**B**


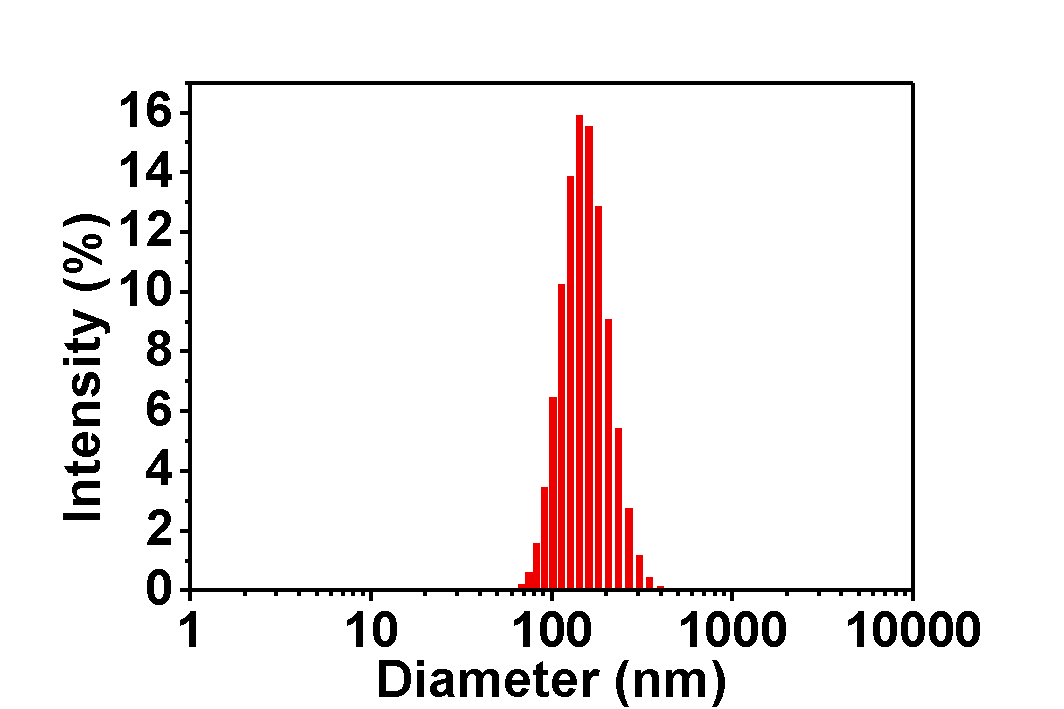


**153.18 nm**

**D**

**Figure S4.** The size of NPs. **(A)**DOX/H20-PLA NPs; **(B)**DOX/H20-PLA@PDA NPs; **(C)**DOX/H20-PLA@PDA-PEG NPs; **(D)**DOX/H20-PLA@PDA-PEG-FA NPs.


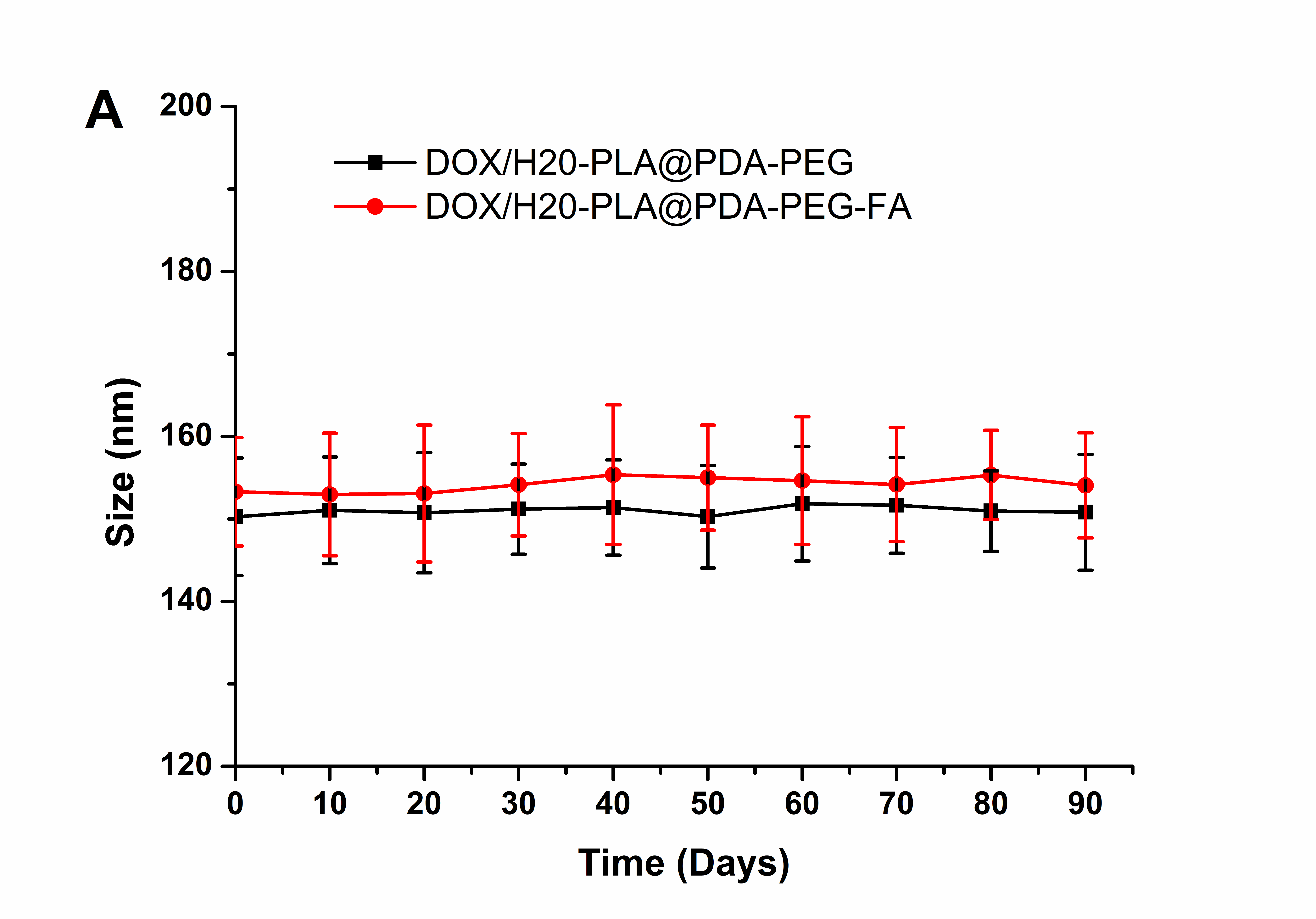

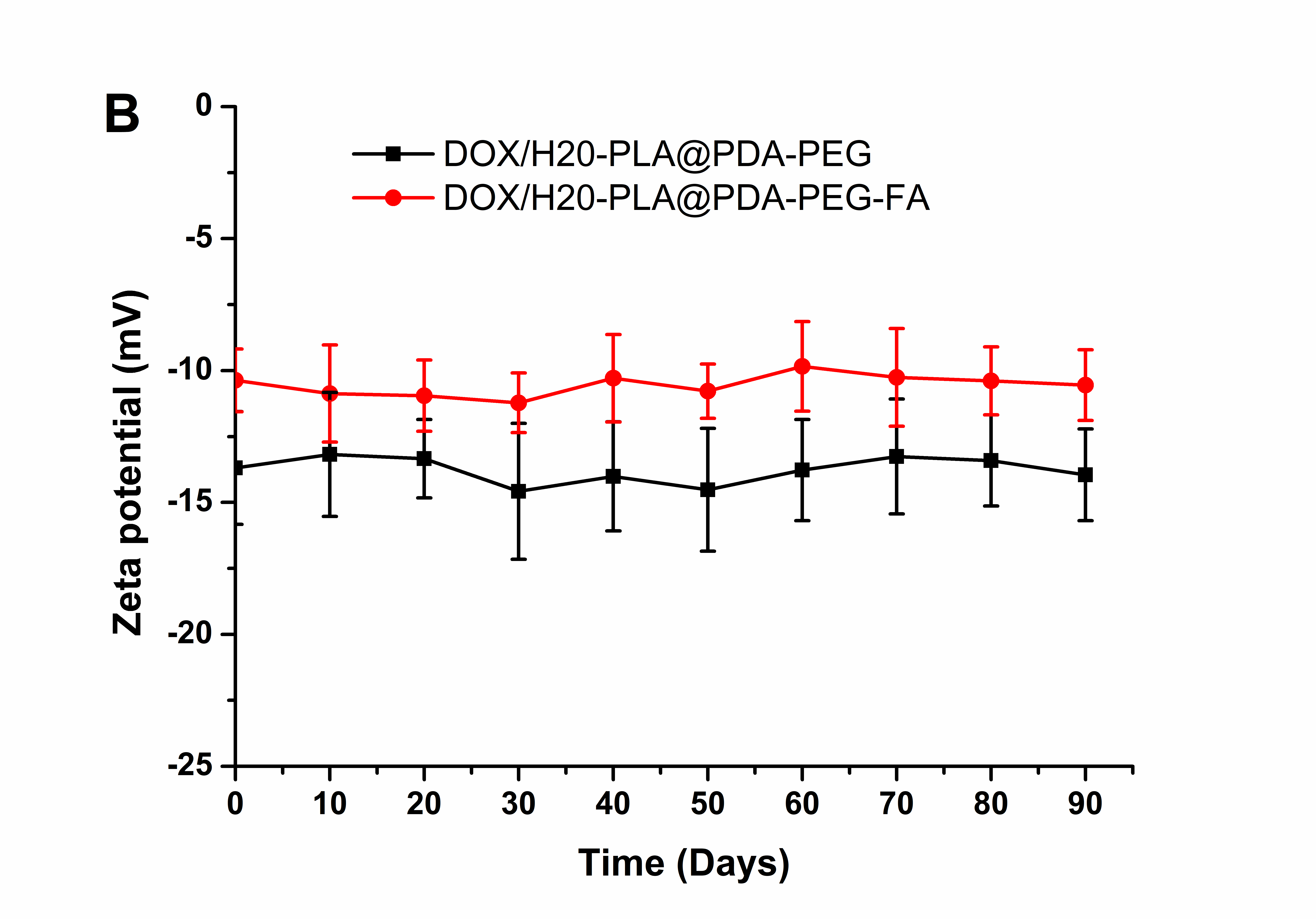


**Figure S5.** The stability of NPs in vitro. **(A)**Particle size and **(B)**zeta potential of DOX/H20-PLA@PDA-PEG NPs and DOX/H20-PLA@PDA-PEG-FA NPs.
